# Effects of DSPP and MMP20 Silencing on Adhesion, Metastasis, Angiogenesis, and Epithelial-Mesenchymal Transition Proteins in Oral Squamous Cell Carcinoma Cells

**DOI:** 10.3390/ijms21134734

**Published:** 2020-07-02

**Authors:** Jaya Aseervatham, Kalu U.E. Ogbureke

**Affiliations:** Department of Diagnostic and Biomedical Sciences, School of Dentistry, University of Texas Health Science Center at Houston, Houston, TX 77030, USA; Jaya.Aseervatham@uth.tmc.edu

**Keywords:** DSPP, MMP-20, lentivirus, EMT, invasion and metastasis, Kallikerins, MMP2, MMP9, integrins, angiogenesis

## Abstract

Recent reports highlight the potential tumorigenic role of Dentin Sialophosphoprotein (DSPP) and its cognate partner Matrix Metalloproteinase 20 (MMP-20) in Oral Squamous Cell Carcinomas (OSCCs). However, the function/mechanism of these roles is yet to be fully established. The present study aimed to investigate the effects of DSPP and MMP20 silencing on specific proteins involved in oral cancer cell adhesion, angiogenesis, metastasis, and epithelial-mesenchymal transition (EMT). Stable lines of DSPP/MMP20 silenced OSCC cell line (OSC2), previously established via lentiviral-mediated shRNA transduction, were analyzed for the effects of DSPP, MMP20, and combined DSPP–MMP20 silencing on MMP2, MMP9, integrins αvβ3 and αvβ6, VEGF, Kallikerin- 4,-5,-8,-10, E-cadherin, N-cadherin, Vimentin, met, src, snail, and Twist by Western blot. Results show a significant decrease (*p* < 0.05) in the expression of MMP2, MMP9, integrin αvβ3, αvβ6, VEGF, Kallikerins -4, -5, -8, -10, N-cadherin, vimentin met, src, snail and twist following DSPP and MMP20 silencing, individually and in combination. On the other hand, the expression of E-cadherin was found to be significantly increased (*p* < 0.05). These results suggest that the tumorigenic effect of DSPP and MMP20 on OSC2 cells is mediated via the upregulation of the genes involved in invasion, metastasis, angiogenesis, and epithelial-mesenchymal transition (EMT).

## 1. Introduction

Oral cancer is the sixth most common cancer in the world with over 600,000 cases diagnosed annually [1]. Over 90% of oral cancers are oral squamous cell carcinoma (OSCC) [2]. Although historic risk factors, including tobacco and alcohol use, continue to be reckoned as etiologic agents, advances in recent years have added infection with oncogenic human papilloma viruses (HPV) to the list of etiologic agents in human OSCC [3]. Surgery, with or without adjunct radiotherapy, remains the mainstay of treatment in patients with primary OSCC because attempts with various chemotherapeutic agents have consistently presented disappointing outcomes. Metastatic dissemination of oral squamous cell carcinoma, which is often a late event, presents significant challenges to the treatment of OSCC patients.

Dentin sialoprotein phosphoprotein (DSPP), a member of the Small Integrin Binding Ligand N-linked Glycoprotein (SIBLING) and its cognate Matrix Metalloproteinase partner (MMP20), is upregulated in several human cancers [4]. A previous study from our laboratory indicates that DSPP upregulation in OSCC and dysplastic oral premalignant lesions correlates with tumor aggressiveness and the transition to OSCC [5]. Furthermore, these studies also showed that DSPP expression at histologically negative resection margins of primary OSCCs correlates with increased recurrence at primary sites [6]. A recent report of the strong enrichment of DSPP at the MMP20 promoter suggests a regulatory role in MMP20 transcription [7]. Further studies show that MMP20/DSPP silencing in OSCC cells resulted in the downregulation of oral cancer stem cell markers and increased sensitivity of OSCC cells to cisplatin treatment. 

Several published reports have also implicated integrins, angiogenic factors, MMPs, several kallikreins, and proteins associated with epithelial-mesenchymal transition (EMT) in tumorigenesis, invasion, and metastasis of OSCC [8,9,10,11,12,13,14,15,16,17]. Here, we investigated the effects of DSPP-MMP20 silencing in the OSCC cell line, OSC2, on the profiles of αvβ3, αvβ6, VEGF, MMP2, MMP9, Kallikerins -4, -5, -8 and -10, E- cadherin, N-cadherin, Vimentin, met, Src, Snail, and Twist. Loss of DSPP and MMP20 decreases invasion, metastasis, angiogenesis, and epithelial mesenchymal transition in OSCC cells. This is with a view to laying the foundation for studies of the mechanisms involved, and the future design of targeted biomimetics based on these mechanisms. 

## 2. Results

### 2.1. Downregulation of MMP2 and MMP9

**Figure 1:**
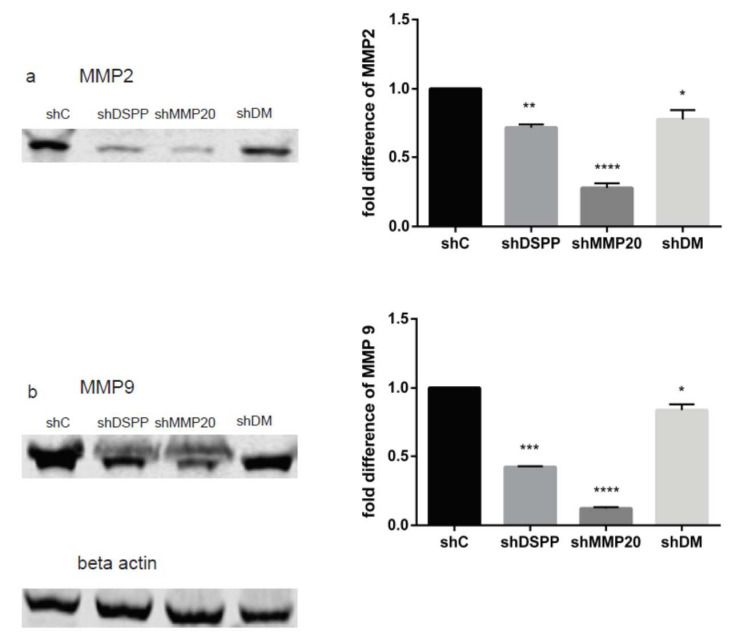
Western blot (WB) analysis of the effect of Dentin Sialophosphoprotein (DSPP) and Matrix Metalloproteinase 20 (MMP20) silencing on MMP2 and MMP9 levels. (**a**) Downregulation of MMP2 in DSPP, MMP20, and combined DSPP was significant (ShDSPP = *p* < 0.01; shMMP20 = *p* < 0.001; shDM = *p* < 0.05) when compared to the control. (**b**) Similarly, downregulation of MMP9 in DSPP, MMP20, and combined DSPP–MMP20 was significant (shDSPP = *p* < 0.01; shMMP20 = *p* < 0.001; shDM = *p* < 0.05) when compared to the control. Beta actin was used as the internal control. Values are given as mean ± SE for 3 independent experiments. shC = Scrambled Control; shDSPP = DSPP Silenced OSC2 cells; shMMP20 = MMP20 Silenced OSC2 cells; DM = Combined DSPP–MMP20 Silenced OSC2 cells; * *p* < 0.05; ** *p* < 0.01; *** *p* < 0.001; **** *p <* 0.0001.

Published reports show that MMP2 and MMP9 are upregulated in OSCCs, where they aid in the invasiveness of oral cancer cells [5,18], and silencing DSPP decreases oral cancer cell invasion [19]. To determine the effect of DSPP and MMP20 silencing on MMP2 and MMP9 levels, OSC2 cells silenced with shRNA for DSPP, MMP20 and both were analyzed by Western blot. As shown in Figure 1, MMP2 (1a) and MMP9 (1b) levels were significantly downregulated in DSPP and MMP20 silenced cells (*p* < 0.01 and *p* < 0.001, respectively) compared with the shRNA controls (shC). Similarly, MMP2 and MMP9 levels were reduced in combined DSPP–MMP20 silenced (shDM) cells (*p* < 0.05). Furthermore, the effects were more apparent when either DSPP or MMP20 were silenced individually, than in combination. This suggests the possibility of compensatory activities between MMP2 and MMP9 when inhibited simultaneously. 

### 2.2. Downregulation of Integrins αvβ3 and αvβ6, and VEGF

**Figure 2:**
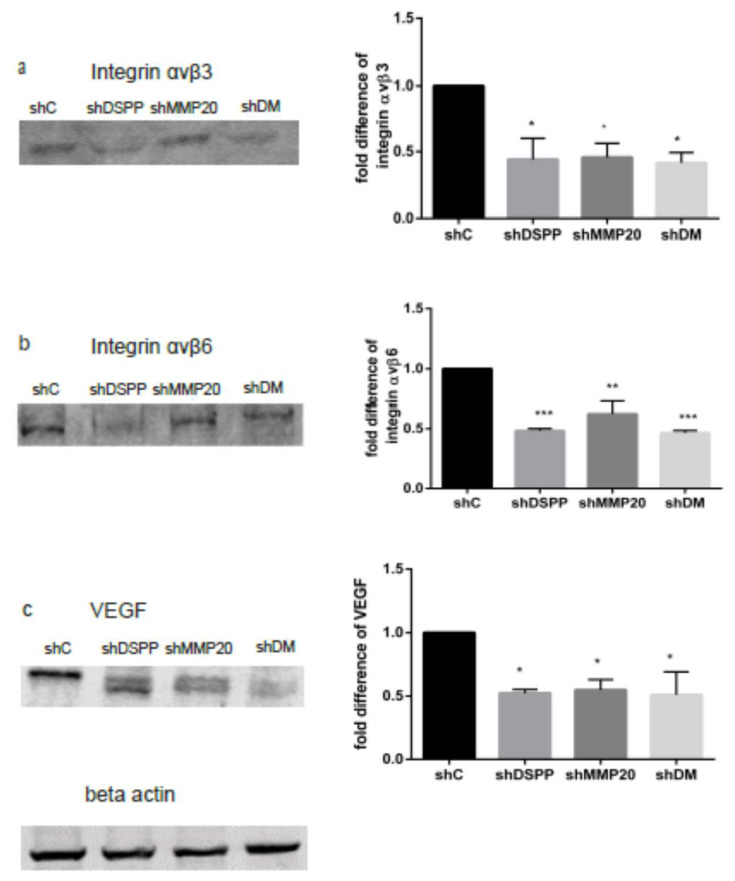
WB analysis of the effects of DSPP and MMP20 silencing on αvβ3, αvβ6, and VEGF levels. (**a**) Downregulation of αvβ3 in shDSPP (50%), shMMP20 (55%), and shDM (45%) were significant (*p* < 0.05) when compared to the control. (**b**) Downregulation of αvβ6 in shDSPP (48%), shMMP20 (38%), and shDM (46%) were significant (*p* < 0.01, 0.001) when compared to the control. (**c**) Downregulation of VEGF in shDSPP (48%), shMMP20 (46%), and shDM (50%) were significant (*p* < 0.05) when compared to the control. Beta actin was used as the internal control. Values are given as mean ± SE for 3 independent experiments. shC = Scrambled Control; shDSPP = DSPP Silenced OSC2 cells; shMMP20 = MMP20 Silenced OSC2 cells; DM = Combined DSPP–MMP20 Silenced OSC2 cells; * *p* < 0.05; ** *p* < 0.01; *** *p* < 0.001.

Studies of OSCCs have shown the upregulation of αvβ3 and αvβ6 in endothelial cells of intra tumoral vasculature and invasive tumor fronts, respectively [11,12]. We and others have reported the upregulation of VEGF in OSCCs [14] and the downregulation of VEGF in DSPP silenced cells [19]. To determine the effect of DSPP and MMP20 silencing in OSC2 cells on levels of αvβ3, αvβ6, and VEGF, Western blot analysis was performed on DSPP, MMP20, and DSPP-MMP20 silenced OSC2 cells. As shown in Figure 2, levels of αvβ3 and αvβ6 were significantly reduced (*p* < 0.05, 0.01, 0.001) following DSPP (shDSPP), MMP20 (shMMP20), and combined DSPP–MMP20 (shDM) silencing when compared to the controls. The αvβ3 level was decreased by 50% in shDSPP, 55% in shMMP20, and 45% in shDM cells (Figure 2a), whereas levels of αvβ6 were decreased by 48%, 38%, and 46% in shDSPP, shMMP20, and shDM, respectively (Figure 2b). Similarly, the VEGF levels were significantly decreased (*p* < 0.05) in shDSPP (48%), shMMP20 (46%), and shDM (50%; Figure 2c). These results suggest that DSPP and its cognate MMP20 partner may modulate the level of integrins αvβ3 and αvβ6, and VEGF in OSCC. 

### 2.3. Downregulation of Kallikreins 4, 5, 8, and 10

**Figure 3:**
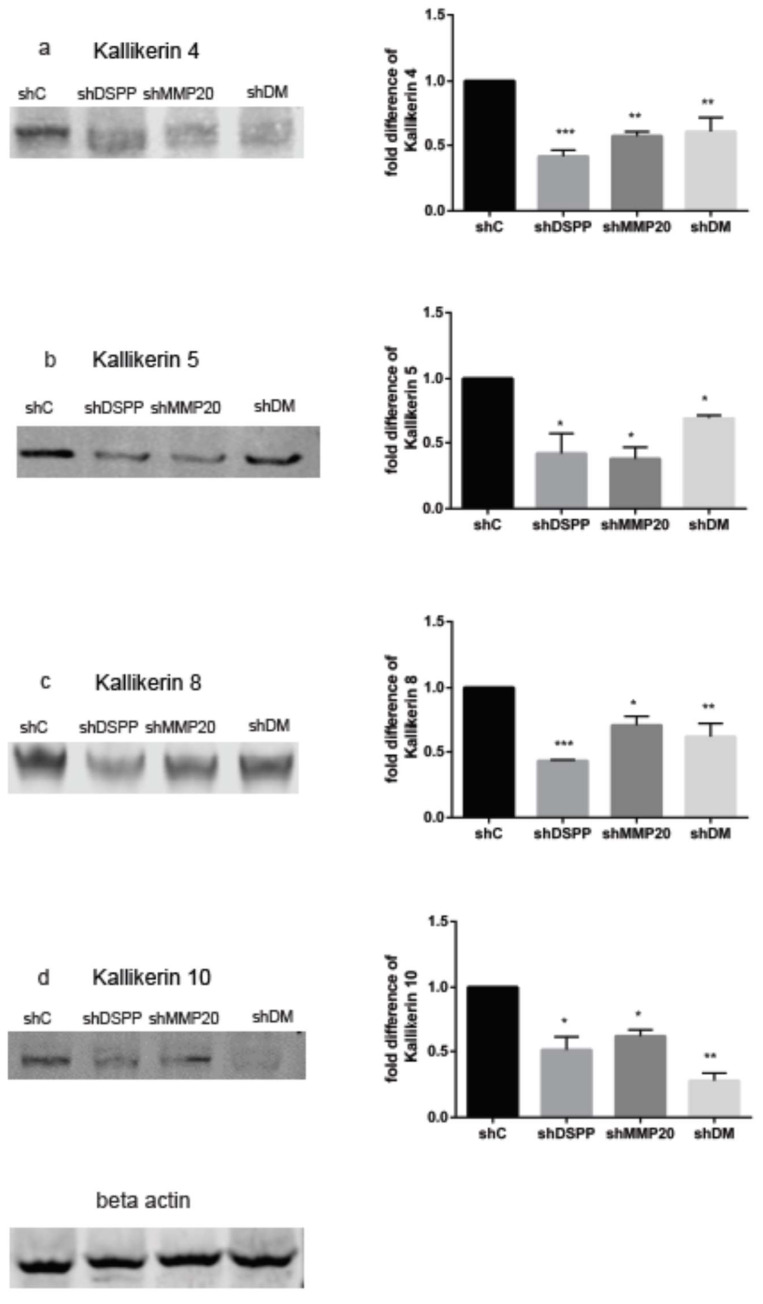
WB analysis of kallikreins levels in DSPP and MMP20 silenced OSC2 cells. (**a**) Downregulation of KLK4 in shDSPP (60%), shMMP20 (40%), and shDM (43%) were significant (*p* < 0.001, 0.01) compared to the control. (**b**) Downregulation of KLK5 in shDSPP (66%), shMMP20 (73%), and shDM (35%) were significant (*p* < 0.05) compared to the control. (**c**) Downregulation of KLK8 in shDSPP (53%), shMMP20 (35%), and shDM (47%) were significant (*p* < 0.001, 0.05, 0.01) when compared to the control. (**d**) Downregulation of KLK10 in shDSPP (48%), shMMP20 (37%), and shDM (73%) were significant (*p* < 0.05, 0.01) when compared with the control. Beta actin was used as the internal control. Values are given as mean ± SE for 3 independent experiments. shC = Scrambled Control; shDSPP = DSPP Silenced OSC2 cells; shMMP20 = MMP20 Silenced OSC2 cells; DM = Combined DSPP–MMP20 Silenced OSC2 cells; *p < 0.05; ** *p* < 0.01; *** *p* < 0.001.

The overexpression of certain kallikreins, notably, KLK4, KLK5, KLK8, and KLK10, in OSCC has been reported [16,17]. To investigate the effects of DSPP and MMP20 silencing on levels of oral cancer-associated kallikreins in oral cancer cells, Western blot analysis was performed on DSPP, MMP20, and combined DSPP–MMP20 silenced OSC2 cells. As show in Figure 3, levels of KLK4, KLK5, KLK8, and KLK10 were significantly reduced when compared to the control, suggesting that DSPP and its cognate MMP20 partner modulate levels of these kallikreins. The KLK4 level (Figure 3a) was decreased by 60% in shDSPP cells (*p* < 0.001), 40% in shMMP20 cells (*p* < 0.01), and 43% in shDM cells (*p* < 0.01) compared to the control. The KLK5 level (Figure 3b) was decreased by 66%, 73%, and 35% in shDSPP, shMMP20, and shDM, respectively (*p* < 0.05). The expression of KLK8 (Figure 3c) was decreased by 53% (*p* < 0. 001) in shDSPP, 35% (*p* < 0. 05) in shMMP20, and 47% in shDM (*p* < 0.01), while levels of KLK10 (Figure 3d) were decreased by 48%, 37%, and 73% in shDSPP, shMMP20, and shDM, respectively (*p* < 0.05, 0.01). 

### 2.4. Levels of Proteins Associated with Epithelial-Mesenchymal Transition (EMT) 

**Figure 4:**
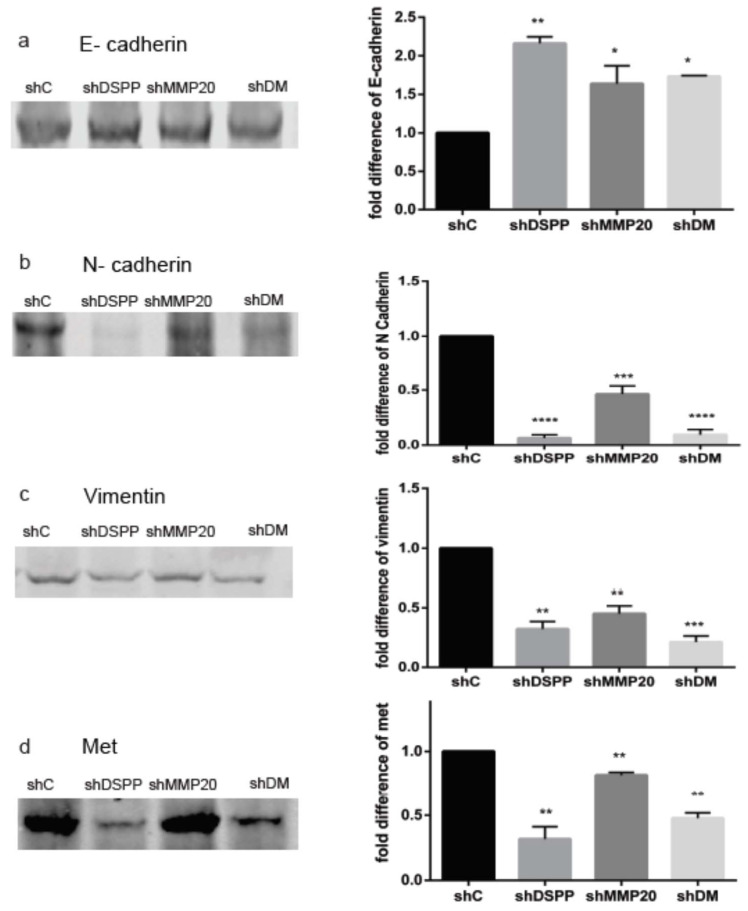

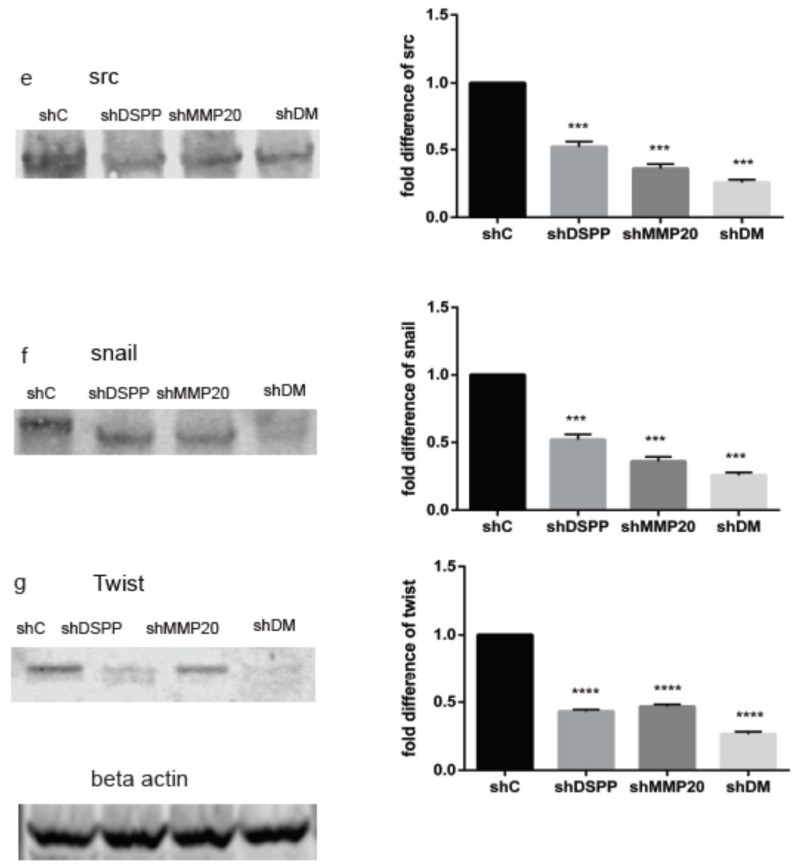
WB analysis of proteins associated with epithelial-mesenchymal transition. (**a**) Increase in level of E-cadherin (2-fold in shDSPP, 1.6-fold in shMMP20, and 1.8-fold in shDM (*p* < 0.01, *p* < 0.05, *p* < 0.05, respectively) when compared to the control. (**b**) Decreased N-cadherin in shDSPP (93%), shMMP20 (54%), and shDM (84%) were significant (*p* < 0.05) when compared to the control. (**c**) Decreased *vimentin* in shDSPP (73%), shMMP20 (55%), and shDM (79%) were significant (*p* < 0.01, 0.001) when compared to the control. (**d**) Downregulation of met in shDSPP (80%), shMMP20 (30%), and shDM (47%) were significant (*p* < 0.01) when compared to the control. (**e**) Downregulation of src (Figure 4e) in shDSPP (60%), shMMP20 (75%), and shDM (85%) were significant (*p* < 0.01) when compared to the control (**f**) Downregulation of snail in shDSPP (46%), shMMP20 (60%), and shDM (74%) were significant (*p* < 0.05) when compared to the control. (**g**) Downregulation of twist in shDSPP (44%), shMMP20 (50%), and shDM (70%) were significant (*p* < 0.001) when compared to the control. Beta actin was used as the internal control. Values are given as mean ± SE for 3 independent experiments. shC = Scrambled Control; shDSPP = DSPP Silenced OSC2 cells; shMMP20 = MMP20 Silenced OSC2 cells; DM = Combined DSPP–MMP20 Silenced OSC2 cells; *p < 0.05; ** *p* < 0.01; *** *p* < 0.001; **** *p* < 0.0001.

Alterations in the levels and activities of several proteins associated with epithelial-mesenchymal transition in the course of OSCC progression have been reported. Western blot analysis was carried out to assess the effects of DSPP, MMP20, and combined DSPP–MMP20 silencing on levels of E-cadherin, N-Cadherin, vimentin, met, src, snail, and twist in OSC2 cells. As shown in Figure 4, the expression of all the above proteins, except for E- cadherin, decreased significantly in shDSPP, shMMP20, and shDM cells when compared to the control. The E-Cadherin level was increased by more than two-fold in the shDSPP cells (*p* < 0.01), 1.6-fold shMMP-20 cells (*p* < 0.05), and 1.8-fold shDM group (*p* < 0.05), when compared to the control (Figure 4a). Conversely, the expression of N-cadherin decreased by 93% for the shDSPP, 54% for shMMP20, and 84% for shDM cells (Figure 4b). Vimentin levels also decreased by 73, 55 and 79 % in shDSPP (*p* < 0.01), shMMP20 (*p* < 0.01), and shDM cells (*p* < 0.001), respectively (Figure 4c). Notably, the effect of silencing was more profound in shDM cells. 

Furthermore, the expression of met (Figure 4d) decreased by 80% in the shDSPP cells, and by 30% and 47% for shMMP20 and shDM, respectively (*p* < 0.05). Src levels (Figure 4e) decreased by 60% in the shDSPP, 75% in shMMP20, and 85% in shDM (*p* < 0.001), whereas levels of snail (Figure 4f) decreased by 46%, 60%, and 74% in the shDSPP, shMMP-20 and shDM cells, respectively (*p* < 0.001). The levels of twist (Figure 4g) decreased by 44% in the shDSPP cells, 50% in shMMP20 cells, and by 70% in the shDM cells (*p* < 0.001). 

## 3. Discussion

The progression of OSCC depends on the accomplishment of established sequential hallmarks of oral carcinogenesis, notably, cancer cell proliferation, invasion, and metastasis. Invasion and metastasis involve the critical steps of cancer cell adhesion, angiogenesis and epithelial-mesenchymal transition (EMT), requiring the activities of integrins, kallikreins, angiogenic factors and proteins implicated in EMT. Our current study investigated the effects of DSPP and MMP20 gene silencing on MMPs (MMP2 and MMP9), integrins (αvβ3 and αvβ6), angiogenic factor (VEGF), Kallikriens (KLK4, KLK5, KLK8, KLK10), and proteins implicated in EMT in OSCCs (E-cadherin, N-cadherin, vimentin, met, src, snail, twist). Our results show that DSPP and MMP20 silencing resulted in the downregulation of αvβ3, αvβ6, VEGF, kallikreins KLK4, KLK5, KLK8, KLK10, N-cadherin, vimentin, met, src, snail, and twist. In contrast, E-cadherin levels were significantly increased following DSPP and MMP20 silencing.

DSPP and several other members of the Small Integrin Binding Ligand N-linked Glycoprotein (SIBLING) family of extracellular matrix proteins have now emerged as significant molecular tools employed by several cancers to facilitate their progression [4]. Other members of the SIBLING family are Bone Sialoprotein (BSP), Dentin Matrix Protein 1 (DMP1), Matrix Extracellular Phosphoglycoprotein (MEPE), and Osteopontin (OPN). Among other shared features, all five members contain the integrin binding RGD motif that enable them to bind to several integrins. These shared features enable the SIBLINGs to mediate cell-matrix interaction and signaling [4]. Furthermore, four members of the family: BSP, DMP1, DSPP, and OPN bind and activate specific MMPs: MMP2, MMP9, MMP20, and MMP3, respectively, in both biochemical and biological systems [18,20,21]. The SIBLING-MMP interaction facilitates surface localization and sequestration of MMPs necessary for extracellular matrix degradation required for the invasion and migration of tumor cells [4].

The upregulation of integrins, αvβ3 and αvβ6, and VEGF in human epithelial cancers, including OSCCs have been reported [11,12,14]. In OSCCs, the expression of αvβ3 is significantly marked in intra tumoral endothelial cells when compared with the level in tumor cells and their stroma [11]. On the other hand, the expression of αvβ6 is notably high in tumor cells at the invasive front [12]. Our previous report demonstrated a significant impediment of the invasion and migration of OSCC cells following DSPP silencing [19]. With our present results, we speculate a mechanism by which DSPP/MMP20 silencing, through its interaction with its cognate MMP20 partner, downregulates αvβ3, αvβ6, and VEGF resulting in decreased invasion and migration of OSCC cells. 

The human kallikrein peptidase (KLK) family of serine proteases, expressed in numerous tissues, plays a crucial role in various physiologic and pathologic processes, including growth and metastasis of malignant tumors, where they have emerged as biomarkers of various cancers [17]. Additionally, the upregulation of a number of KLKs, notably -4, -5, -8, -10, in oral cancers has been reported [16,17]. Published reports indicate that KLK4 facilitates the spread of cancer cells via basement membrane and connective tissue degradation [16]. Furthermore, reports show that KLK4 silencing inhibited the invasion and migration of OSCC cells, while also regulating the epithelial-mesenchymal transition (EMT) process, via the PI3K/AKT signaling pathway [9]. With respect to KLK5, published reports show that the overexpression of KLK5 in normal oral mucosal epithelium results in disaggregation of keratinocytes, whereas the silencing of KLK5 in OSCC cells increased cell–cell cohesion (aggregation) [13]. Overall, these results indicate that KLK5 enhances cancer cell metastasis via a mechanism involving cleavage of desmoglein1 (Dsg1) and the consequent loss of cell–cell junctional integrity [13]. 

Although KLK8 and KLK10 overexpression in OSCC and other cancers have been associated with tumor aggressiveness and overall prognosis, their precise mechanisms of activity are yet to be clearly deciphered. Our results show that DSPP/MMP20 silencing results in a significant downregulation of KLK4, KLK5, KLK8, and KLK10 in OSC2 cells. The present data reflect varying degrees of KLK suppression with DSPP, MMP20, and combined DSPP–MMP20 silencing (Figure 3). Notably, silencing DSPP resulted in a significantly higher suppression of KLK4 and KLK8, than with MMP20 and combined DSPP–MMP20 silencing. The reverse appears to be the case with KLK10 suppression, where the effects of combined DSPP–MMP20 silencing was more profound than with DSSP or MMP20 silencing (Figure 3). With respect to KLK5, there was no significant difference in level of suppression following DSPP, MMP20, or combined DSPP–MMP20 silencing (Figure 3). 

Given these results, it is logical to postulate that the downregulation of these KLKs in OSCC, following DSPP/MMP20 silencing, results in the impediment of basement membrane breakdown and cell–cell disaggregation; the steps necessary to decrease cancer cell invasion and metastasis. It is worthy to note that our previous study showed that DSPP silencing resulted in the decreased invasion and migration of OSC2 cells [19]. Furthermore, it is plausible that other hallmarks of oral carcinogenesis, including cancer cell proliferation and angiogenesis, significantly reduced following DSPP silencing [19], may also implicate mechanisms involving KLK downregulation. Further studies beyond the scope of the present one will aim to decipher any DSPP–MMP20–KLK interactive mechanisms in oral carcinogenesis.

While the process of EMT is essential for driving plasticity during embryonic development, it is deemed incidental behavior of cancer cells during carcinogenesis. In epithelial cancers, EMT enables cancer cells to trade part of their epithelial morphology for partial mesenchymal phenotype [10]. This tradeoff facilitates epithelial cancer cell interaction with their matrix environment: an interaction ultimately necessary to accomplish the cancer cell phenotypic hallmark of invasion and metastasis [10]. In epithelial cancers, including OSCCs, EMT results in the downregulation of epithelial markers such as E-cadherin, and the upregulation of mesenchymal markers, including N-cadherin and vimentin [8,15]. In oral carcinogenesis, there is a progressive and significant reduction in the level of E-cadherin through the various grades of epithelial dysplasia to OSCC [8]. In contrast, the levels of N-cadherin increased with the progression of oral epithelial cells from dysplasia to invasive OSCC [8]. Our current data, showing significantly increased levels of E-cadherin and significantly decreased levels of N-cadherin, vimentin, and EMT transcription factors (EMT-TFs: met, src, snail, twist) following DSPP/MMP20 silencing, suggest that the role of these indicia/markers of EMT in OSCC is dependent on DSPP/MMP20 activity. 

As indicated above, in addition to N-cadherin and vimentin, the EMT-TFs: met, src, snail, and twist, were similarly down regulated following DSPP/MMP20 silencing in our present study. While EMT-TFs promote cancer progression (invasion, metastasis, and drug resistance), they do not play a role in tumorigenesis [15,20,21,22]. Published reports of the mechanistic network and interplay between these proteins provide a framework for studying their hierarchical interactions with DSPP/MMP20 in oral cancer progression, particularly invasion and metastasis. Met, a receptor tyrosine kinase, binds to its ligand hepatocyte growth factor (HGF) to trigger phosphorylation of the receptor and kinase activity [23]. In cancer cells, this interaction facilitates invasion via increased production and activity of MMP-1, -3, -9, and VEGF [22]. HGF also upregulates snail, which in turn suppresses the expression of E-cadherin, thereby promoting EMT [24].

Src, a member of the src family of kinases, is upregulated in OSCC, characteristically at the connective tissue–tumor interface [25]. Src may be activated by adhesion receptors, G proteins, and receptor tyrosine kinase, and suppresses E-cadherin [26]. By activating the cas adaptor protein, AP-1, src modulates the upregulation of MMP2 and MMP9, which are important players in OSCC metastasis [27]. Similarly, snail inhibits the expression of E-cadherin through the SMAD pathway, increases the expression of MMP9, increases tumor angiogenesis, and promotes cell survival via the MAPK and PI3K pathways [28,29]. The upregulation of twist in OSCC is reported to partially account for the repression of E-cadherin [30]. Twist also activates the AKT pathway, increases MMP2, MMP9, and VEGF production, while inhibiting apoptosis via the activation of Bcl2 [31]. The effects of twist contribute to the sum of effects of upregulation of mesenchymal EMT markers, vimentin, fibronectin, N-cadherin, and the cell adhesion protein periostin [32]. In summary, the decreased expressions of the EMT-TFs (met, src, snail, and twist), following DSPP/MMP20 silencing in our present study, allow for the upregulation of E-cadherin and the downregulation of MMP9, VEGF, N-cadherin, and vimentin. Significantly, EMT-TF decreased expression may combine to prevent the partial EMT required to facilitate tumor cell invasion and metastasis.

## 4. Materials and Methods 

Appropriate Institutional Review Board approval for this study was obtained from the University of Texas Health Science Center at Houston.

### 4.1. Cell Line and Culture Conditions

OSC2 cell line was purchased from American Type Culture Collection and was cultured in DMEM/F12 medium containing 10% FBS (Cal. #A13708; Gibco, Grand Island, NY, U.S.A.), supplemented with 1% Penicillin/Streptomycin and 500 ng/mL Hydrocortisone (Cal. #H0888-1G; MilliporeSigma, Burlington, MA, U.S.A.), and maintained in the presence of 5% CO_2_ humidified air at 37 °C.

### 4.2. Stable Lines of DSPP/MMP 20 Silenced OSC2 Cells

Recently, our laboratory established and published stable lentiviral mediated DSPP, MMP20, combined DSPP–MMP20 silenced OSC2 cell lines with scrambled (ShC) counterparts as controls [33]. ShRNA Plasmid A also used as negative control was obtained from Santa Cruz Biotechnology (cat. #sc-108060; Santa Cruz, CA, USA). The stably silenced phenotype of these cell lines was validated (75% silencing) [33], cultured as a monolayer in DMEM/F12 medium containing 10% FBS (Invitrogen, Carlsbad, CA, USA), supplemented with 1% Penicillin/Streptomycin and 500 ng/mL Hydrocortisone (Sigma Aldrich, St. Louis, MO, USA), and maintained in the presence of 5% CO^2^ humidified air at 37 °C. 

### 4.3. Western Blot

Total cell lysates were prepared from each group (control, shDSPP, shMMP-20, and shDM) and 50 µg of protein was resolved on SDS–PAGE gel using the Mini-Protean Tetra Cell unit (Biorad, Hercules, CA, U.S.A) and transferred to a polyvinylidene (PVDF) membrane (Millipore; Burlington, MA, U.S.A). The membrane was blocked with 5% milk for an hour and the primary antibodies were added and incubated overnight. The primary antibodies for MMP 2 (sc 8835), MMP- 9 (sc 8835), VEGF (sc57469), snail (sc-28199), and Src (sc-32789) were purchased from Santa Cruz biotechnology. E-cadherin was purchased from invitrogen (ma1-06300). Vimentin (5741) and N-cadherin (13116) from cell signaling, twist (ab50581), Integrin αvβ6 (ab124968), Kallikrein 4 (ab71234), Kallikrein 5 (ab176299), Kallikrein 8 (ab150395), Kallikrein 10 (ab172094), and β-actin (ab4970) were purchased from Abcam. Integrin αvβ3 (bs1310R) was purchased from Bioss. The membrane was washed thrice with TBST, incubated with their respective secondary antibodies from Licor, and signal was detected using the infrared LI-COR Imaging system (LI-COR Biosciences; Lincoln, NE, U.S.A). Quantification of the proteins was performed using actin as the internal control. The protein expression is given as a fold difference with respect to control.

### 4.4. Statistical Analysis

All the experiments were performed in triplicates and data were expressed as mean ± SE. Statistical analysis was performed using SigmaStat Version 3 (Systat software, Point Richmond, CA, USA) and SAS 9.1 (SAS Institute, Inc., Cary, NC, USA). Due to the skewed nature of the data, the Kruskal–Wallis test and Dunn multiple comparisons were used any time more than two groups were compared. The criterion for significance was *p* < 0.05, *p* < 0.01, and *p* < 0.001 for the study.

## 5. Conclusions

The accomplishment of the essential steps of invasion and metastasis in the furtherance of oral carcinogenesis requires critical steps involving cancer cell adhesion, angiogenesis, and epithelial-mesenchymal transition (EMT). Here, we provide data showing that DSPP and MMP20 silencing resulted in downregulations of αvβ3 and αvβ6 (adhesion), VEGF (angiogenesis), kallikreins KLK4, KLK5, KLK8, KLK10 (invasion and metastasis), N-cadherin, vimentin, met, src, snail, and twist (EMT). In contrast, the E-cadherin level was significantly increased following DSPP and MMP20 silencing. Our data provide the foundation for future studies of the interplay and mechanisms of DSPP/MMP20 interactions with integrins, angiogenic factors, and proteins implicated in EMT. This is with a view to deciphering therapeutic strategies that may target the mechanistic networks involved in the treatment of OSCC.

## Abbreviations

HPV	human papilloma viruses
OSCC	Oral Squamous Cell Carcinoma
MMP	Matrix Metalloproteinase
VEGF	Vascular Endothelial Growth Factor
EMT	Epithelial Mesenchymal Transition
DSPP	Dentin Sialophosphoprotein
